# Curing neurophobia in medical schools: evidence-based strategies

**DOI:** 10.3402/meo.v21.32476

**Published:** 2016-09-27

**Authors:** Abdelrahman Ibrahim Abushouk, Nguyen Minh Duc

**Affiliations:** 1 Faculty of medicine, Ain Shams University, Cairo, Egypt; 2 NovaMed Medical Research Association, Cairo, Egypt; 3 Faculty of Public Health, University of Medicine and Pharmacy, Ho Chi Minh City, Vietnam

**Keywords:** medical students, neurophobia, neurology, solutions, education

## Abstract

Medical students often perceive neurology as the most difficult medical specialty. This perception is described as ‘neurophobia’ in the medical literature. Several studies have cited poor teaching, complex examination, and separation of basic and clinical sciences as major factors in the development of neurophobia. These negative perceptions can have serious implications, such as decreasing the students’ desire to consider neurology as a future career and increasing referrals from other specialists to avoid dealing with neurological conditions. Faced with increasing demands of healthcare systems and the global burden of neurological conditions, there is a rising need for further research and innovative strategies to improve students’ perceptions of clinical neurology. This review discusses evidence-based recommendations and educational interventions to cure neurophobia in medical education.

As defined by Jozefowicz in 1994, neurophobia is ‘the fear of neural sciences and clinical neurology that originates from the students’ inability to apply their basic science knowledge to clinical practice leading to paralysis of thinking or action’ (1). Several multinational studies have investigated students’ attitudes toward neurology teaching and practice, focusing on the risk factors and possible solutions (2–6). Considering the results of these studies, neurophobia appears to be a global phenomenon that affects students in various stages of medical education (6).

In a recent study by Fantaneanu et al. (3), the factors contributing to neurophobia were classified into modifiable and non-modifiable groups. The non-modifiable risk factors include students’ past exposure to neurology on clinical, personal, and educational levels, while modifiable risk factors include poor teaching, complex terminology, separation of basic science teaching and clinical application, lack of standardized patients with known neurological illness during simulation sessions, and the stigma that neurologists are unsatisfied with their careers (2–6). Despite having no scientific data supporting his claim, Jozefowicz stated that neurophobia is a disease that starts early in medical school and reaches its peaks during basic and clinical neuroscience courses. He also claimed that it affects about 50% of medical students with an equal gender incidence (1).

The implications of neurophobia are usually under-recognized in medical education. According to the World Health Organization, neurological diseases constitute about 6.3% of global morbidity and contribute to about 12% of global mortality (7). These numbers are only expected to grow as the population ages. Facing these increasing numbers, there is a shortage of supply of clinical neurologists to the healthcare system (8). Neurophobia is believed to negatively influence the choice of medical students against selecting neurology as a future career. Moreover, primary care physicians tend to avoid dealing with neurological conditions, resulting in increasing numbers of referrals to neurology clinics (9).

A systematic review by McCarron (10) concluded that the existing body of literature regarding interventions in neurology education is deficient, and that there is a need for high-quality research to develop strategies to tackle this problem. This article discusses nine possible solutions to cure neurophobia (Table 1), highlighting evidence-based recommendations and educational interventions to help students overcome their fear of clinical neurology.

**Table 1:** Nine strategies for curing neurophobia in medical schools

Implement team-based learning strategy in educational sessions using small group discussion, teamwork, and immediate feedback. Combine the hypothesis-driven and pragmatic screening approaches into teaching neurological examination. Optimize the use of three-dimensional simulators, video recordings, and online resources in teaching neurological examination. Expand the allocated settings for clinical teaching and mandate student training in outpatient clinics. Integrate basic and clinical sciences through problem-based learning approach and sustain reinforcement of basic concepts during clinical years. Recruit standard patients to neurological examination teaching sessions and train them to provide both verbal and written feedbacks to trainees. Reform clinical neurology curricula to reflect the health priorities of the community, achieve social values, and encourage lifelong learning. Build a positive reputation for neurology and neurologists using key researchers and disseminate knowledge regarding recent advances in neurology. Conduct further research on neurology education and practice to introduce new educational interventions into neurology teaching.

## Implement team-based learning strategy in educational sessions

Team-based learning (TBL) is a student-centered instructional strategy that incorporates small group discussion, team work, and immediate student feedback in the educational process (11). Through these activities, TBL enhances students’ interactions while maintaining the instructor's control during the delivery of the educational material (12). Recently, two randomized controlled trials have compared TBL to other conventional methods of teaching as lectures and problem-based learning (PBL) and they concluded that students who attended TBL sessions had higher confidence, less neurophobia, and achieved higher scores on the post-test exam (13, 14). This was further confirmed in a case series by Balslev (15), in which students exposed to small group discussions had higher diagnostic accuracy in approaching neurologic patients.

Practically, TBL comprises three stages: 1) preparing students through explanation of the session's objectives; 2) assessment of students’ preparation on the individual level (Individual Readiness Assurance test) and the team (Team Readiness Assurance test); and 3) providing a clinical scenario for group discussion under instructor's supervision to direct the students’ clinical decisions in the appropriate way. Finally, immediate feedback from students regarding achievement of the session's objectives and the effectiveness of the teaching strategy is obtained and documented (11, 12). As stated by Michaelsen et al. (16), constructing a TBL session must satisfy four goals listed as proper formation of the groups, holding students accountable of their individual and group work, promotion of both learning and team development, and frequent feedbacks from the learners (16).

## Combine the hypothesis-driven and pragmatic screening approaches into teaching neurological examination

Most medical schools teach students to perform a pragmatic screening approach, comprising all elements of neurological examination to elicit all possible clinical findings (17). This is often performed during Objective Structured Clinical Examinations (OSCE) to test the student's ability to perform a comprehensive neurological examination (18). Besides the cognitive difficulty and higher time consumption of this approach, failing to recall and document all diagnostic findings in the broad neurological examination is more likely (19). Hypothesis-driven examination follows an iterative approach, in which students formulate diagnostic hypotheses based on minimal clinical findings from the patient's history, and then gather more information from examination to support or reject these hypotheses (20).

In a randomized controlled trial by Kamel et al. (18), students were randomized to perform either a checklist-based neurological examination on a group of patients with a history-based provisional diagnosis (hypothesis testing), or a traditional screening examination. Students in the hypothesis-testing group had a higher likelihood of eliciting more abnormal signs in a slightly shorter examination time.

However, depending solely on this approach increases the liability of missing important clinical findings that may indicate a significant pathology within the nervous system as a separate condition, or as a part of a clinical syndrome (17, 20). Moreover, it increases the possibility of misinterpreting normal findings as abnormal to support the initially claimed diagnosis (18). Therefore, a combination of both hypothesis-driven and screening examinations may work better than a single approach in enhancing students’ clinical and cognitive skills (17).

## Optimize the use of three-dimensional simulators, video recordings, and online resources in teaching neurological examination

e-Learning, defined as ‘the use of internet technologies to enhance knowledge and practice’, is often used to deliver educational material through distance learning and to supplement classroom learning (21). Utilizing digital technology to formulate 3D computer simulators of the nervous system can make the experience of learning neuroanatomy more comprehensible and enjoyable (2). Although some elements of the neurological examination are better understood through hands-on experience, such as testing muscle tone and power, a considerable part of the neurological examination can be taught by viewing video clips of professional examination (22). This approach maximizes the students’ neuroscience experience and allows for formulating and testing abstract concepts (23).

Two randomized controlled trials showed that using computerized interactive tutorials and 3D simulation techniques were associated with higher post-test scores, better visualization of concepts, and more preference of these methods over conventional lectures (24, 25). These results were further confirmed by several case series which showed that the use of computer-aided neuroanatomical programs and videotaped vignettes improved clinical examination scores, covered the shortage of patients available for education, and decreased the students’ fear of clinical neurology (22, 26, 27). Therefore, in institutions with lack of resources to recruit standard patients, designing virtual electronic patients may satisfy this purpose.

The wide availability of high-tech devices, such as smart phones, tablets, audio response tools, social media and podcasts, can contribute to adopting these technologies in neurology education (28). The current educational infrastructure should be manipulated to allow introduction and use of these technologies (29). Moreover, their use should be balanced with traditional teaching methods according to the students’ needs to optimize the delivery of the educational content (30).

Online resources can present medical data in different formats, ranging from plain text to fully interactive platforms (31). Greater access to these resources can supplement patient exposure and bedside teaching with lower costs (5). The existing literature suggests that online resources can only complement rather than replace conventional teaching techniques through improving the accessibility of educational materials (32). The capabilities of online resources can extend beyond providing educational materials, to helping physicians reach a clinical diagnosis (33). A group of residents showed that Google can lead to the correct diagnosis up to 50% of diagnostic results upon the time when encountering challenging clinical cases (34).

In a randomized controlled study by Lewis et al. (35), providing a group of students with a web-based neuroanatomical localization program was associated with higher post-test scores in the intervention group. Moreover, in a published case series by Lim et al. (36), designing a web-based game called ‘Neurolocalization Game; NLG Box 1’ in which students are provided with a case scenario of a virtual patient, including details of the patient's history and clinical examination findings, was associated with a higher confidence in reaching a diagnosis and more satisfaction compared to conventional learning techniques.

Incorporating online teaching methods in neurology education still faces many obstacles, such as lack of maintenance of distance learning websites and lack of the trained manpower to manage these tools (31). Developing solutions to overcome these drawbacks can enhance the role of these techniques in the educational process.

## Expand the allocated settings for clinical teaching and mandate student training in outpatient clinics

Several studies have cited poor teaching as a major contributor to the development of neurophobia in medical students. When students were surveyed about the possible ways to improve neurology education, they highlighted increasing the amount of planned teaching as a primary solution to achieve this goal (3–6).

This can be achieved by extending the durations of neurology rotations, providing more opportunities for extracurricular learning, and expanding the settings in which clinical teaching is delivered to include all health resources of the community, such as hospital wards, outpatient clinics, rehabilitation units and ambulatory care facilities. Moreover, the contribution of all types of health care providers, including primary care physicians and nurses should be utilized and appreciated. Utilizing medical practice resources and personnel in medical education can help students link both theoretical and practical aspects of clinical education (37).

Dividing the educational experience of clinical medicine into separate rotations for different specialties contributes to viewing the nervous system and its pathologies as a distinct branch of medicine with more complex rules than other branches (28). Integration of neurology education into other areas of medicine may help reduce that perceived gap by medical students. Moreover, bridging neurology with epidemiological and behavioral sciences can help students develop a sense of the importance of neurological principles for their future careers in medicine (37).

Since the 1970s, neurologists have reported a disconnection between the clinical conditions encountered during training and those encountered in practice (38, 39). Health professionals attribute this to the inability of inpatient hospital rotations to provide the optimal training to manage outpatient conditions (38). Therefore, spending more time in the outpatient clinic as a part of the students’ neurology rotation is highly recommended.

This strategy will allow students to gain more exposure to neurologic patients and acquire stronger skills to identify the common presentations of neurological disorders (33). Moreover, the confidence built through patient contact may contribute to lessening the students’ fear of neurological practice. Participating in reasoning how a patient's diagnosis is reached may help to eradicate the sense of ambiguity of neurological conditions (40).

During training in the outpatient clinic, students should not only be passive observers but should be allowed to experience patient contact, especially with patients with known Neurological conditions (41). This approach would fulfill the Experiential Learning Cycles (ELC) educational model, in which the learner's subjective experience is of critical importance to the learning process (40).

## Integrate basic and clinical sciences through PBL approach and sustain reinforcement of basic concepts during clinical years

As defined by Jozefowicz, neurophobia originates from the inability to apply basic science knowledge to clinical situations (1). Several cross sectional studies have cited the deficiency of basic science background as a barrier to learning in clinical neurology (3–6).

According to Jozefowicz, an effective program that integrates basic and clinical neuroscience may increase the students’ motivation to learn the workings of the nervous system and overcome neurophobia (1). This can be achieved through integration of clinical scenarios during basic science education through PBL, as well as mandating basic science education in conjunction with neurology rotations. These techniques have been found to improve recall in neurology and clinical medicine generally (42, 43). Moreover, this integration would allow medical students to view the basic science information from a clinical perspective, helping them to focus on the essential and clinically relevant information (1).

In a randomized controlled study by Heckman et al. (42), students were randomized either to learn basic neuroscience in a conventional manner through lectures and conferences or through PBL. Students in the PBL group had significantly higher scores at their final exams than the conventional group. Two controlled cohort studies found a beneficial effect for replacing conventional neurology courses with a pilot integrated neuroscience course in improving the students’ scores and attitudes toward neurology (44, 45).

In a study of Canadian medical students, higher comfort levels with neurology were reported, as well as more confidence that they acquired the necessary knowledge for their future practice when surveyed at the end of their neurology block. However, this confidence tended to decrease as students progressed to the next year of medical school (3). This finding highlights the importance of constant exposure to neurology education throughout medical school education, preventing neurophobia from leaking into the students’ perception of clinical neurology.

This strategy can be fulfilled by disseminating neurology education sessions in different curricula along the whole length of medical school and is termed ‘distributed practice’. It has been shown to provide a sustained positive attitude and a more competent performance in neurological examination, even after 14 months from the last educational intervention (46).

This sustained exposure should not stop at graduation, and further resources must be allocated to provide continuing medical education (CME) in clinical neurology throughout a physician's professional life (37). This can be achieved through professional conferences, training sessions, and online CME courses, which have been shown to increase physicians’ factual knowledge and adherence to guidelines (47).

## Recruit standard patients to neurological examination teaching sessions

Despite the technological advances in medical education and the ability to design virtual patients with proposed neurological conditions, some elements of the neurological examination, such as eliciting muscle tone and power are still better palpated than simply visualized (22). Standard patients are chosen based on their medical history and often trained to reliably portray or recall their experience at medical encounters. They can also be trained to provide patient-centered feedbacks regarding the examinee performance and behavior (48).

In a controlled cohort study by Safdieh et al. (49), students who were assigned to perform neurological examination on standard patients achieved higher OSCE scores than their peers who did not have access to standard patients. In a randomized controlled study by Park et al. (50), students who received both verbal and written feedbacks from standard patients achieved higher post-test scores than those who received written feedbacks only.

However, using standard patients may require more resources as skilled patients usually get higher payment and it raises ethical questions regarding the physical and psychological health of these patients (48). Following examination, students should get immediate feedbacks from these patients in both written and verbal forms.

## Reform clinical neurology curricula to reflect the health priorities of the community and achieve social values

An ideal neurology curriculum should fulfill certain criteria: 1) stratify its contents according to their importance for the practicing physician; 2) reflect the local health priorities and focus on the common and preventable conditions; and 3) ensure building of professional competence and social values, not only data retention.

To assist with the stratification of contents, various neurology associations have developed clinical curricula containing a ‘must know guidelines’ for medical students and young physicians (41). These guidelines indicate that medical students should learn how to perform an initial evaluation for patients with neurological complaints and to recognize when referral for further consultation is needed (51).

Establishing a central educational unit at each medical school with the authority to modify the curriculum according to the health needs of the community can ensure that the curriculum reflects local priorities. The curriculum should focus on the conditions which are common and preventable or which present as emergencies (37). It should also highlight the resource availability within the community, and the national economic status, to provide the best health care service within these settings (8).

Finally, the neurology curriculum objectives should extend beyond the cognitive retention of information. The relationship between neurological disorders and mental illness engages the patient's personal values and the ethical principles of the community in the management of these conditions. Physicians must be aware of the local cultural traditions, and their assessment should consider the cultural and ethnic diversity of patients, as well as matters related to gender (37).

## Build a positive reputation for neurology and neurologists

Several studies have shown that students believe that neurologists are generally unsatisfied with their profession, and that neurology ranks lower than other specialties in terms of financial reward and contribution to their patients’ lives. These beliefs have been found as early as the first year of medical school, suggesting that these preconceptions originate before admission into medical school (3).

These negative beliefs can be eradicated through building role models and establishing positive reputations for both institutions and individuals based on scholarly productivity. Exceptional researchers and practicing physicians are marketable. Recent evidence suggests that professional development of teaching and assessment staff can reverse the students’ negative attitudes toward neurology (52).

Moreover, the current advances in the practice of clinical neurology and the significant contributions by neurologists to the quality of life of their patients should be advocated and shared with the public. During medical school, students should be granted the opportunity to carry out research projects in neurology-related topics, meet inspiring neurologists, and receive additional teaching beyond the course curriculum (53).

## Conduct further research on neurology education and practice

In a systematic review by McColgan et al. (54), the existing body of literature discussing neurology education was marked as deficient with increasing needs to introduce more effective educational interventions into the teaching of neurology. Therefore, further research should be conducted to develop educational strategies to reduce neurophobia.

Conducting a high-impact educational study would require: 1) collaboration between medical educators, practicing physicians, and certification boards; 2) more contribution of funding resources to support innovative educational research; and 3) communicating the findings of research to stakeholders and directors of medical education facilities (55).

Future research should address several considerations such as providing student-centered strategies while maintaining the best available patient outcome, and integration of the recent diagnostic and therapeutic advances in developing new educational strategies to help students keep pace with the ongoing evolution of clinical practice (56).

## Conclusion

This article presents nine evidence-based recommendations to address the students’ negative perceptions toward clinical neurology. It offers stakeholders and directors of medical education facilities a summary of the most successful educational interventions in the field of neurology education. More evidence-based strategies and novel approaches should be introduced to meet the increasing global burden of neurological disorders. Therefore, future educational research is a top priority to improve neurology education.
